# Bibliometric analysis of tumor necrosis factor in post-stroke neuroinflammation from 2003 to 2021

**DOI:** 10.3389/fimmu.2022.1040686

**Published:** 2022-10-27

**Authors:** Yang Zhao, Qihan Zhu, Chen Bi, Jichao Yuan, Yujie Chen, Xiaofei Hu

**Affiliations:** ^1^Department of Basic Medicine, Third Military Medical University, Army Medical University, Chongqing, China; ^2^Department of Graduate, China People’s Police University, Langfang, China; ^3^Department of Neurology, Southwest Hospital, Third Military Medical University, Army Medical University, Chongqing, China; ^4^Department of Neurosurgery, Southwest Hospital, Third Military Medical University, Army Medical University, Chongqing, China; ^5^State Key Laboratory of Trauma, Burn and Combined Injury, Southwest Hospital, Third Military Medical University, Army Medical University, Chongqing, China; ^6^Department of Radiology, Southwest Hospital, Third Military Medical University, Army Medical University, Chongqing, China

**Keywords:** neuroinflammation, stroke, TNF, bibliometric analysis, VOSviewer, CiteSpace

## Abstract

**Objective:**

Tumor necrosis factor (TNF), a crucial cytokine, has important research value in post-stroke neuroinflammation (PSN). We analyzed the studies that have been conducted in this area and used bibliometric methods to predict research hotspots and identify trends regarding TNF in PSN.

**Methods:**

Publications were accessed at the Science Citation Index Expanded 1975-2021 (SCI expanded), Web of Science Core Collection (WoSCC), on May 1, 2022. Additionally, software such as CiteSpace and VOSviewer were utilized for bibliometric analyses.

**Results:**

In total, 1391 original articles and reviews on TNF in PSN published from 2003 to 2021 were identified. An upward trend was observed in the number of publications on TNF in PSN. These publications were primarily from 57 countries and 1446 institutions, led by China and the United States with China leading the number of publications (NP) and the US with the number of citations (NC). The League of European Research Universities (LERU) and Journal of Neuroinflammation, respectively were the most prolific branches and journals. Zhang, John H. published the most papers and Finsen, Bente had the most cited papers. One paper by Kettenmann, H. published in 2011 reached the highest level of Global Citation Score (GCS). The keyword co-occurrence and reference co-citation analyses suggest that poststroke therapy and potential mechanistic pathways are important topics related to PSN in recent years. Reference burst detection suggests new burst hotspots after 2015, focusing on pathway modulation and discovery of therapeutic targets, suggesting a substantial development in the study of TNF in PSN research.

**Conclusion:**

The present bibliometric analysis shows a continuous trend of increasing literature related to TNF in PSN, and shows that TNF plays an important role in PSN involves multiple immune mechanisms and may contribute as a potential target for neuroprotective therapeutics after stroke. Prior to 2011, most of the research was focused on discovering the specific role of TNF in PSN, and in recent years studies have mainly targeted the exploration of the signaling pathways. Future research prospects may lie in finding key therapeutic targets in pathway of TNF in PSN.

## Introduction

Cerebrovascular disease has become a disease with a high mortality and disability rate worldwide (1). Stroke disease, one of the most significant cerebrovascular diseases, has a rising trend in incidence in recent years at a younger age, imposing a huge economic burden on the lives of patients and society (2). One of the primary reasons for the poor prognosis of stroke disease is post-stroke neuroinflammation (PSN) which is caused and exacerbated by the microglia-mediated inflammatory response (3). This response is an important pathological mechanism through which large amounts of TNF-α are released in ischemic stroke, thus worsening PSN. The TNF-α, one of the most important inflammatory cytokines in the TNF family (4), has a vital central mediating role in the regulation of PSN (5). It is a key element of the neuroinflammatory response linked with several neurological disorders. According to some strong evidence, TNF-α expression is associated with stroke injury and stroke recovery (6–8). What’s more, focusing on effectively improving the development of neuroinflammation by regulation of TNF expression, is likely to find a way forward in repair and recovery after stroke (9, 10).

The current research focusing on the direction of TNF in PSN has been continuously improved and explored. However, there is no complete and objective assessment of the publication output, institutions, influential countries, authors and their collaborations, research progress and directions, hot spots, and frontiers of this study.

As an interdisciplinary discipline, bibliometrics allows the analysis and study of the systems and characteristics of the literature, using statistical and mathematical methods to quantify all knowledge carriers and understand the foundations and frontiers of the field of study (11, 12). The most obvious advantage is that it is a secondary visualization of previous research on the topic and can be used to identify changes the research focus related to the topic over time through cluster analysis, keyword analysis, reference analysis and other analysis methods, as well as to predict future research directions (13, 14).For example, in Yu’s outstanding work, they used a citation network analysis to determine the trajectory of intuitionistic fuzzy sets, as a new attempt to provide a useful guide for our bibliometric analysis (13). What’s more, the future progress of the research topic can be characterized and predicted by comparing the contributions of different countries, institutions, authors, and publications (15). There are already scholars performing bibliometric analysis in many medical fields of research, such as tinnitus (16), mesenchymal stem cells (17), and novel coronavirus pneumonia drugs (18).

In this study, the publications were analyzed and research directions of TNF in studies related to PSN from 2003 to 2021 were assessed and the corresponding network mapping was carried out using VOSviewer and CiteSpace software. These findings provide knowledge support and direction for basic research and clinical treatment applications in this research area.

## Methods

### Data sources and search strategies

**Figure 1:**
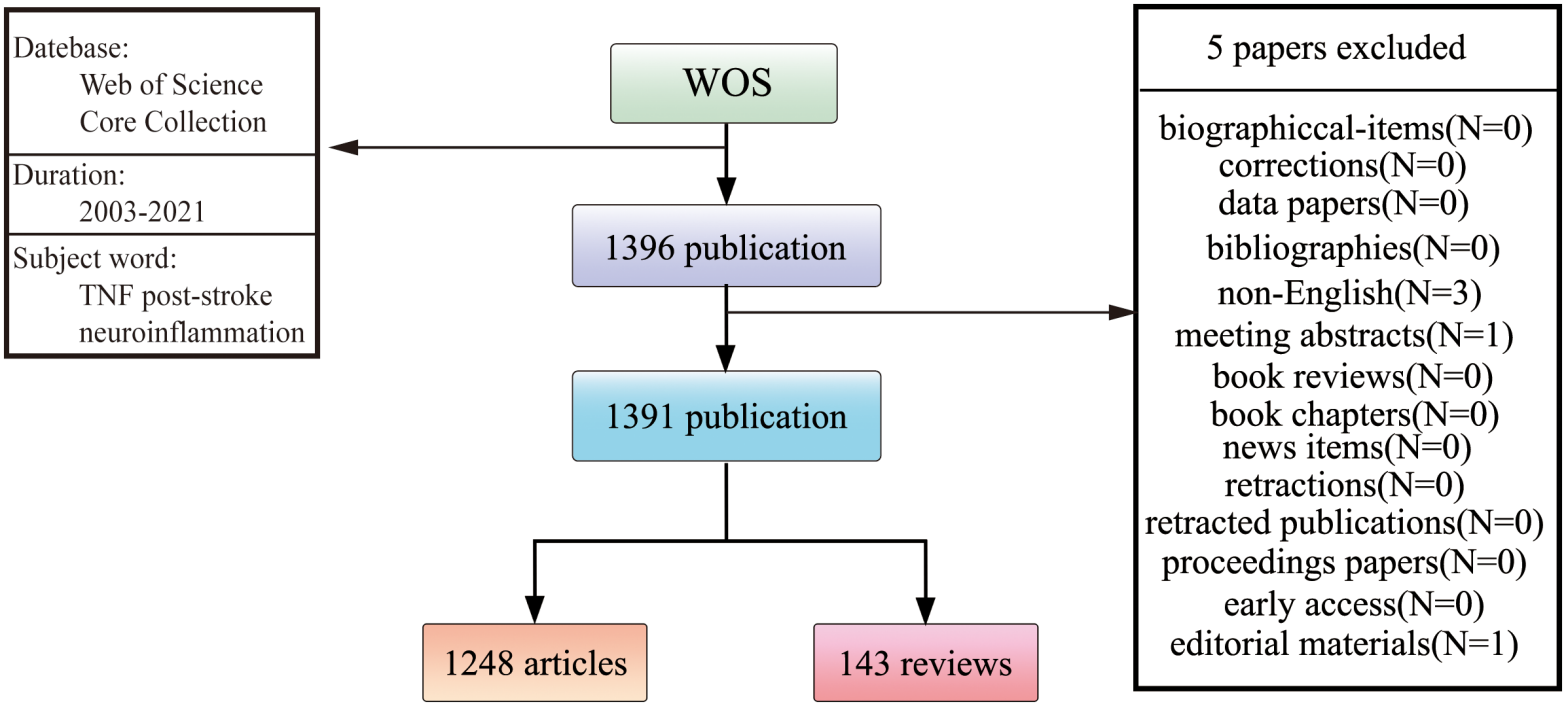
Screening flow chart.

This study collected data from the Web of Science Core Collection (WoSCC), and the time span of the search was set between 2003 and 2021 and was conducted on May 1, 2022. The search terms were as follows: ts=(“stroke” OR “intracerebral hemorrhage” OR “ischemic stroke “ OR “brain infarction” OR “brain stem infarction” OR “cerebral infarction” OR “cerebral stem infarction” OR “ischemic encephalopathy” OR “infarction encephalopathy” OR “brain ischemia” OR “brain stem ischemia” OR “cerebral ischemia” OR “cerebral stem ischemia”) and (TS=(“TNF” OR “Tumor Necrosis Factor”)) and (TS=(“neuroinflammation” OR “microglia” OR “microglia activation”)). Among the various forms of relevant publications (published papers, reprints, book chapters and reviews, conference abstracts, news items, letters, editorial material, corrections, data files, early access, bibliographies, and biographical entries), the English articles and reviews were the ones analyzed. In total, 1248 articles and 143 reviews were accessed and studied. The strategy for accessing and retrieving articles and reviews has been elaborated as well (**Figure 1**).

### Bibliometric analysis

The text data were obtained on WoSCC, and bibliometric indicators such as the NP and the number of non-self-citations (NC) were extracted from these text data representing the quality of publications. In particular circumstances, the H-index is utilized to assess the published content of a region or country, a journal, or an institution as well as the scholarly achievement of an individual (19). The statistical computing and graphics were done by employing VOSviewer, CiteSpace, and Microsoft Office Excel 2019. The data collected from WoSCC was utilized to create bibliometric maps and visualized as well as analyzed for potential information by employing VOSviewer and CiteSpace respectively. Microsoft Office Excel 2019 was employed to plot statistical charts.

## Results

### Research profile

According to the search strategy (**Figure 1**), 1391 papers and reviews published from 2003 to 2021 were retrieved, containing a total of 59,524 citations, with an average of 45.1 citations per paper. The H-index for all publications was 111.

### Annual trends in the number of publications

**Figure 2:**
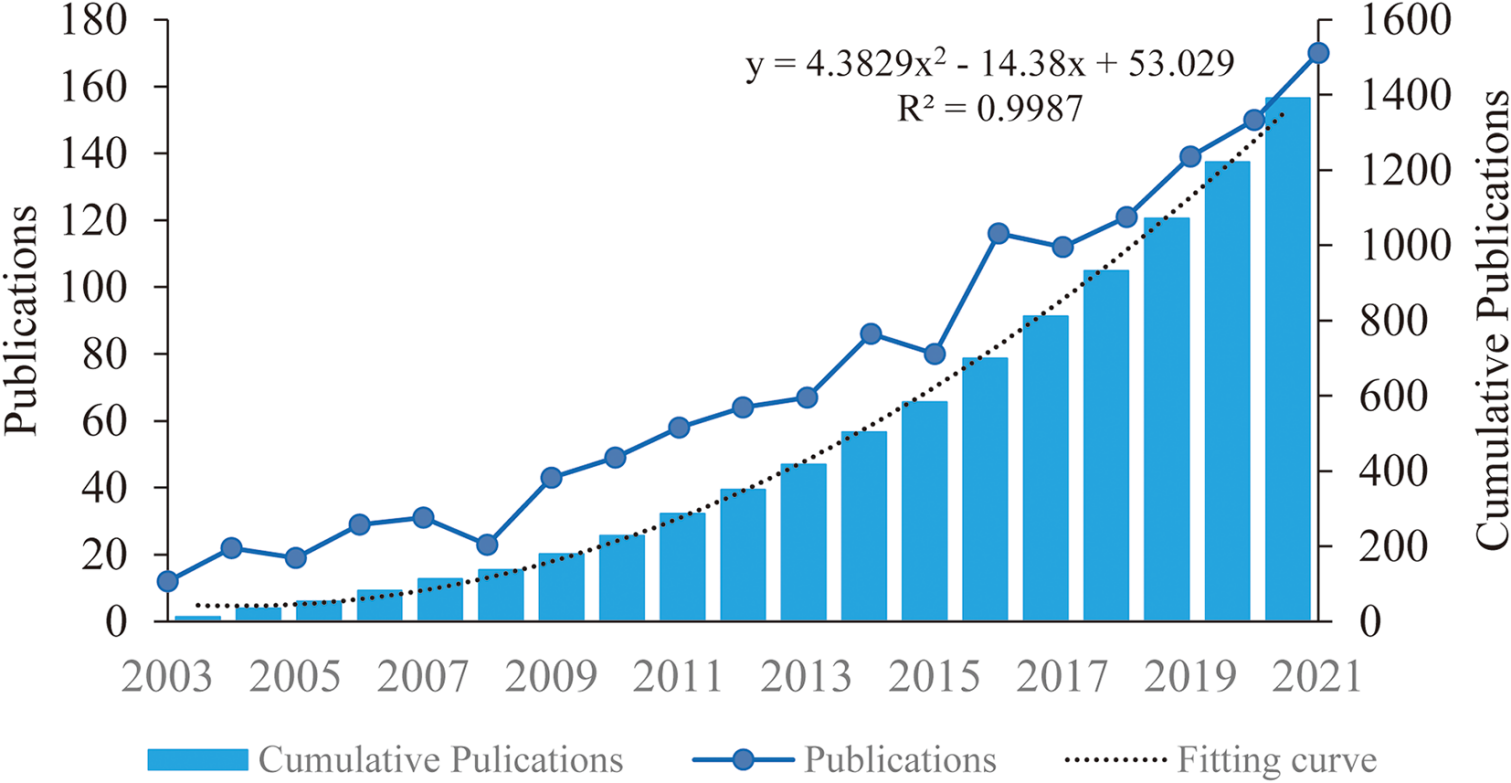
Publication growth trend of TNF in PSN publications from 2003 to 2021.

The change in the number of annual publications characterizes the pace and advancement of research on this topic and the level of attention paid to this area regarding research (20). There were 1391 annual publications on TNF in PSN between 2003 and 2021 (**Figure 2**). The number of publications on this topic tended to increase from 2003 to 2021, with a slight decrease in 2005, an increase from 2009 to 2014, and a decrease in 2015 and 2017. The number of publications exceeded 150 in 2020 and 2021. The curve of the total number of publications fits the quadratic function curve with a goodness of fit R^2^ of 0.9987, indicating that the total number of publications will grow more rapidly in the future.

### Country analysis

**Figure 3:**
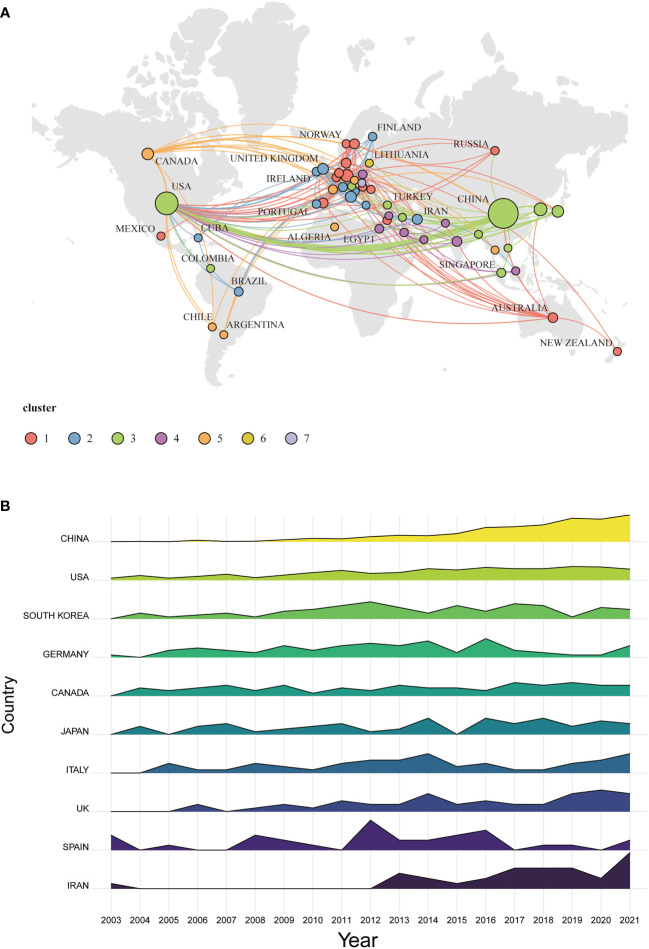
Country analysis. **(A)** Inter-country cooperation network map. The size of the nodes indicates the amount of publications, and the thickness of the lines represents the intensity of cooperation. **(B)** ridgeline plot.

The analysis of publications from different countries can indicate to some extent the importance placed on the research area by the country as well as the degree of influence of that country. The VOS clustering indicates the division of the inter-country cooperation networks into seven clusters of which five are cooperative network clusters, while LITHUANIA and SLOVAKIA countries do not cooperate with other countries (cluster #6 and cluster #7, respectively). Among the different cooperative network clusters, inter-country cooperation is generally low, except for cluster #3, where closer cooperation is shown by China and the United States suggesting that countries should further deepen their cooperation to promote the development of the discipline (**Figure 3A**). The ridgeline plot represents the relative trends in a number of publications per year for the top 10 countries. To enhance the visualization, after normalization (each country’s annual issuance divided by its overall national issuance separately) it represents that China’s research in this area has been on an increasing trend since 2003, the US annual issuance has been relatively stable, and after 2012, the major research countries have increased their research momentum (**Figure 3B**). These findings suggest that the interest in the study of TNF in PSN has become widespread which has kickstarted a rapidly developing field. As shown in **Table 1**, China had the highest number of publications (652), followed by the USA (361) and South Korea (82). A major portion of the total citations (42.74%) was comprised of the 25,441 times cited US papers that preceded China (19,331) and Germany (6,572). Additionally, the U.S. had the top-ranked H-index (84), which was twofold higher than South Korea (33) and Canada (30). The UK was ranked at the top in terms of the average citations (120.84) with Germany (99.58) occupying the second position, demonstrating the high quality of publications in both countries. The relatively low average citations in China, South Korea, and Iran indicates the need for improved quality of publications in these countries.

**Table 1:** Top 10 most productive countries.

Rank	Country	Np	%of (1,391)	Nc	H-index	Average citations
1	CHINA	652	46.87	19331	65	29.51
2	USA	361	25.95	25441	84	66.52
3	SOUTH KOREA	82	5.90	2997	33	36.55
4	GERMANY	66	4.74	6572	37	99.58
5	CANADA	58	4.17	4203	30	73.74
6	JAPAN	57	4.10	4278	25	75.05
7	ITALY	48	3.45	2775	26	57.81
8	UK	43	3.09	5196	24	120.84
9	SPAIN	31	2.23	2634	23	84.97
10	IRAN	30	2.16	682	16	22.73

### Institutional and author analysis

**Figure 4:**
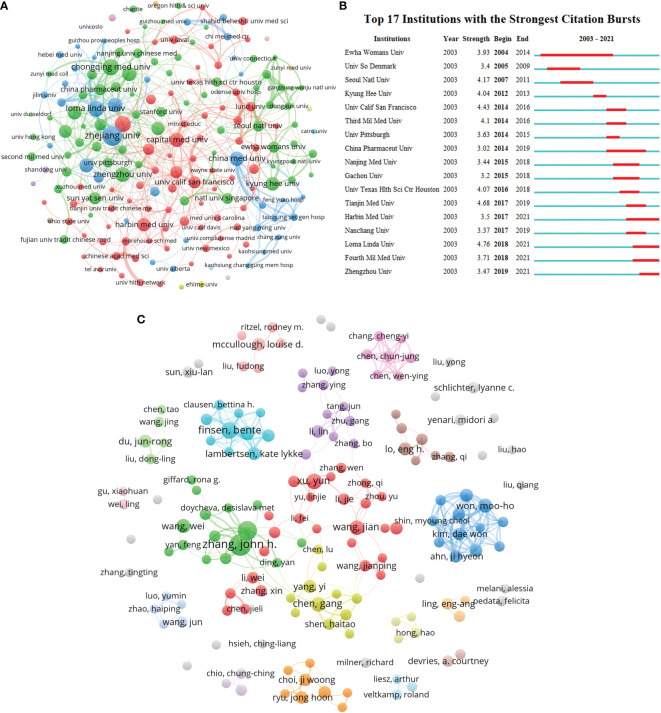
Visualization of institutions and authors. **(A)** Inter-institutional collaboration analysis **(B)** Top 17 institutions in terms of burst detection, where red bars indicate the burst year. **(C)** Collaboration analysis among authors.

The units with the most publications related to TNF in PSN research are listed in **Table 2**. The European Research University Consortium with the most publications (57), citations (4477), and H-index (32), preceded the Zhejiang University and the University of California system. Additionally, in terms of average citations, the University of Texas system was at the top (92.17) with the University of California system (86.88), and the League of European Research University (78.54) following closely. In addition, 50% of the top 10 affiliated institutions are from China and 40% are from the United States. The institutional collaboration co-occurrence map shows weak collaborative relationships among institutions, suggesting the need for greater collaboration (**Figure 4A**). The 17 affiliations with the most representative outbreak intensity were also illustrated (**Figure 4B**). Loma Linda University has the highest outbreak intensity. The 10 topmost published authors, with 116 papers, accounted for 8.34% of the total number of papers (**Table 3**). They had 5454 citations, accounting for 9.16% of the total citations. Zhang, John H. from Loma Linda University ranked first in this topic research area, followed by Tang, Jiping from Loma Linda University and Finsen, Bente from University of Southern Denmark. As depicted in **Table 3**, Finsen, Bente had the highest average citations (95.62). Additionally, the percentage of the 10 topmost authors from the USA was 40% while 30% were from China, and the author’s co-occurrence network was also explored (**Figure 4C**).

**Table 2:** Top 10 most productive affiliations.

Rank	Affiliations	Country	Np	Nc	H-index	Average citations
1	LEAGUE OF EUROPEAN RESEARCH UNIVERSITIES	EUROPE	57	4477	32	78.54
2	ZHEJIANG UNIVERSITY	CHINA	36	1008	19	28.00
3	UNIVERSITY OF CALIFORNIA SYSTEM	USA	34	2954	24	86.88
4	NANJING UNIVERSITY	CHINA	28	846	16	30.21
5	CHONGQING MEDICAL UNIVERSITY	CHINA	27	475	13	17.59
6	US DEPARTMENT OF VETERANS AFFAIRS	USA	27	1846	20	68.37
7	LOMA LINDA UNIVERSITY	USA	26	731	16	28.12
8	ZHENGZHOU UNIVERSITY	CHINA	25	551	14	22.04
9	SHANGHAI JIAO TONG UNIVERSITY	CHINA	24	698	13	29.08
10	UNIVERSITY OF TEXAS SYSTEM	USA	23	2120	16	92.17

**Table 3:** Top 10 most productive authors.

Rank	Author	Country	Np	Nc	H-index	Average citations
1	Zhang, John H	USA	22	620	14	28.18
2	Tang, Jiping	USA	17	428	10	25.18
3	Finsen, Bente	DENMARK	13	1243	12	95.62
4	Xu, Yun	CHINA	11	194	6	17.64
4	Chen, Gang	CHINA	10	296	9	29.6
6	Deierborg, Tomas	SWEDEN	9	620	9	68.89
7	Wang, Jian	CHINA	9	823	9	91.44
8	Won, Moo-Ho	SOUTH KOREA	9	110	6	12.22
9	Lo, Eng H	USA	8	647	7	80.88
10	McCullough, Louise D	USA	8	473	7	59.13

### Journal analysis

The VOSviewer software was used to count the published journals related to the study of TNF in PSN to explore and identify the most prominent and productive journals. In total, the number of publications published in 367 well-reputed academic journals comes to 1391 publications. The highest ranking journals regarding the publication of related articles were depicted in **Table 4** with the Journal of Neuroinflammation (87 publications, IF: 9.587, JCR:Q1) occupying the top position and the Journal of Cerebral Blood Flow and Metabolism (37 publications, IF: 6.96, JCR:Q1) along with the Neuroscience (33 publications, IF: 3.708, JCR:Q3) following close behind. An analysis of the journals from which the references originated shows the contribution of each journal to the knowledge base of the field. Of the 4340 cited journals, two journals had cited more than 3,000 times. The journals as depicted in **Table 4** were ranked highest to lowest with Stroke (citations: 3,982, IF: 10.17, JCR:Q1) preceding the Journal of Cerebral Blood Flow and metabolism and the Journal of Neuroscience.

**Table 4:** The top 10 most productive journals and cited journals.

Rank	Journals	Np	IF and JCR division (2021)	H-index	Cited journals	Citation	IF and JCR division (2021)	H-index
1	Journal of Neuroinflammation	87	9.587, Q1	38	Stroke	3982	10.17, Q1	15
2	Journal of Cerebral Blood Flow and Metabolism	37	6.96, Q1	30	Journal of Cerebral Blood Flow and Metabolism	3193	6.96, Q1	30
3	Neuroscience	33	3.708, Q3	23	Journal of Neuroscience	2824	6.709, Q1	13
4	Brain Research	30	3.61, Q3	18	Brain Research	2111	3.61, Q3	17
5	Experimental Neurology	30	5.62, Q2	18	Journal of Neuroinflammation	1767	9.587, Q1	38
6	Plos One	30	3.752, Q2	18	Glia	1684	8.073, Q1	15
7	Molecular Neurobiology	29	5.682, Q1	17	Journal of Neurochemistry	1664	5.546, Q2	18
8	Journal of Neurochemistry	27	5.546, Q2	17	Proceedings of the National Academy of Sciences of the United States of America	1620	12.779, Q1	3
9	International Immunopharmacology	21	5.714, Q2	15	Neuroscience	1416	3.708, Q3	23
10	Neurochemistry International	21	4.297, Q2	15	Journal of Immunology	1360	5.426, Q2	4

**Figure 5:**
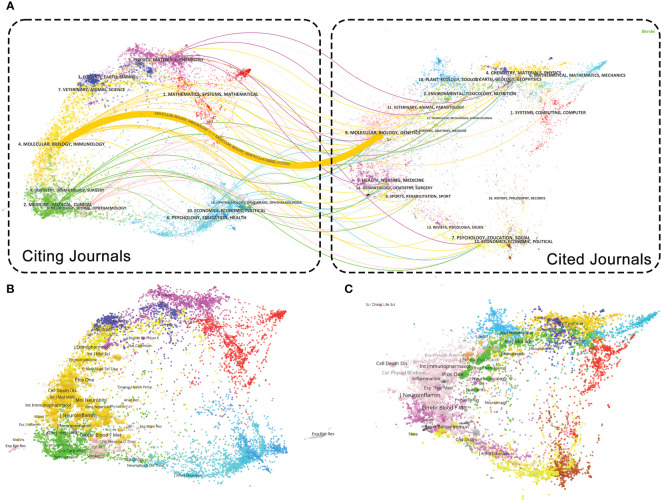
Journal overlay map. **(A)** Dual map overlay of journals. The citing journals collection is on the left, the cited journals collection is on the right, and the colored paths indicate the citation relationships; **(B)** Citing journals overlay; **(C)** Cited journals overlay.

The dual-map overlay analysis fabricated by Chen and Leydesdorff L. reveals patterns in the scientific mix of global scientific journal maps. A dual map of studies on TNF in PSN published between 2003 and 2021 was generated (**Figure 5A**). All colored curves originating from the citing journal collection map (left panel) and pointing to the cited journal collection map (right panel) indicate the path of citation links. The citing journal overlay and cited journal overlay maps generated on the Global Scientific Journal Map for all detailed journal information published on this topic between 2003 and 2021 were explored (21), respectively (**Figures 5B, C**). The results of the journal biplot overlay indicate a relatively high concentration of journals studied on this topic. The source journals of the cited literature and references are mainly in the field of Molecular/Biology/Genetics, and the citation chain is mainly generated in this field, with less cross-field research. In the future, the fields of Physics/Materials/Chemistry, Veterinary/Animal/Science, Medicine/Medical/Clinical, Neurology/Sports/

Ophthalmology, and Psychology/Psychology/Health are likely to be emerging areas. Education/Health are likely to be emerging frontier research areas.

### Reference analysis

The CiteSpace’s co-citation analysis of references is a core feature of this program (22). CiteSpace’s co-citation network of references extracts cluster labels based on the cited literature, and the citing and cited literature represents the research frontier and knowledge base (23). Therefore, the analysis of typical clusters can help in understanding the core information regarding this area of research as well as the evolution and development of research regarding TNF in PSN. A scaling factor k=25 was set, and 1391 cited literature references with certain influences were extracted using g-index that allowed the identification of homogeneous clusters of literature that was cited more frequently and was associated with TNF in PSN research.

**Figure 6:**
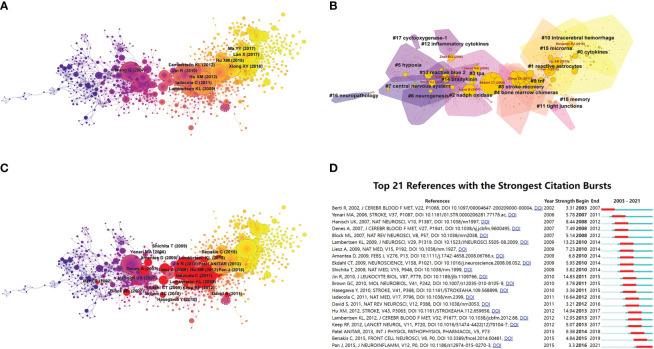
Visualization of co-citation and clustering network, where the nodes in the figure represent citations and the links between nodes represent co-citation relationships. **(A)** Co-citation analysis of references; **(B)** Clustering network analysis of references; **(C)** Burst detection of co-citations, where red represents bursts; **(D)** Burst detection of top 21 references, where red bars indicate burst years.

The co-citations of the references were also examined and illustrated such that the labels showed the first author with the ten most cited references and the year (**Figure 6A**). The aforementioned references were done by Iadecola, C. et al. They published the original article entitled “The immunology of stroke: from mechanisms to translation” (24) in Nature Medicine in 2011 and another article entitled “Commensal microbiota affects ischemic stroke outcomes by regulating intestinal γδ T cells” (25).

The structure of the knowledge and the research boundaries of the area of interest can be examined utilizing cluster analysis. The cluster analysis of the co-cited literature summarizes the areas researched in the field and explores the hot spots and research directions (26). The largest 19 clusters extracted from the references of 1391 cited articles were thoroughly examined (**Figure 6B**). The noun phrases usually constitute the cluster labels and are extracted from the titles of the cited articles using the log-likelihood ratio (LLR) algorithm, such as #0 cytokines, #1 reactive astrocytes, #2 NADPH oxidase, #3 tpa, #4 bone marrow chimeras, #5 hypoxia, #6 neurogenesis, #7 central nervous system, #8 stroke recovery, #9 tnf, #10 intracerebral hemorrhage, #11 tight junctions, #12 inflammatory cytokines, #13 reactive blue 2, #14 bradykinin, #15 microRNA, #16 neuropathology, #17 cyclooxygenase-1, #18 memory (**Figure 6B**). The total Q value was 0.8123 and the average profile of the weights for each cluster was 0.8618, indicating reasonable cluster quality. The purple contours represent early clusters, such as #6 neurogenesis and #16 neuropathology, while the yellow contours represent the most recent cluster labels, such as #0 cytokines and #10 intracerebral hemorrhage. The node size is proportional to the mediator centrality size, and the mediator centrality measures the likelihood of any shortest path through a node in the network (**Figure 6B**). The nodes with high intermediary centrality may be located between two clusters or in the middle of some clusters, serving to connect different clusters or articles within clusters and facilitating shifts in research topics and research paradigms, etc. (intermediary) (27, 28). The item that is positioned at the top by centrality is Ekdahl CT (2009) in Cluster #3, with a centrality of 0.38. The second one is Xiong XX (2011) in Cluster #3, with a centrality of 0.28. The third is Ajami B. (2007) in Cluster #2, with a centrality of 0.21. The fourth is Pan J (2015) in Cluster #0, with a centrality of 0.19. The fifth is Hu XM (2015) in Cluster #0, with a centrality of 0.17. The sixth is Gelderblom M. (2009) in Cluster #3, with a centrality of 0.15. The seventh is Clausen B.H. (2005) in Cluster #13, with a centrality of 0.14. The eighth is Babcock AA (2006) in Cluster #2, with a centrality of 0.13. The ninth is Yenari MA (2006) in Cluster #14, with a centrality of 0.13. The tenth is Lambertsen K.L. (2012) in Cluster #9, with the centrality of 0.12.

The burst detection was used to reveal sudden increases in popular citations over time that were illustrated such that nodes indicate references, and nodes with red circles indicate burst citations in this region (**Figure 6C**). The details of the 21 most burst citations were examined (**Figure 6D**). The item positioned at the top by bursts is Iadecola C. (2011) in Cluster #4, with bursts of 16.64. The second one is Hu XM (2012) in Cluster #4, with bursts of 14.94. The third is Jin R. (2010) in Cluster #8, with bursts of 14.83. The fourth is Lambertsen K.L. (2009) in Cluster #4, with bursts of 13.25. The fifth is Lambertsen K.L. (2012) in Cluster #9, with bursts of 12.95. The sixth is Patel ANITAR (2013) in Cluster #1, with bursts of 9.38. The seventh is Hanisch U.K. (2007) in Cluster #2, with bursts of 8.44. The eighth is Denes A (2007) in Cluster #3, with bursts of 7.49. The ninth is Liesz A. (2009) in Cluster #3, with bursts of 7.23. The tenth is Amantea D. (2009) in Cluster #3, with bursts of 6.80. It is worth noting that the literature of Pan J (2015) in cluster #0 remains hot and continues even to this day. These hot papers suggest that the knowledge base of TNF in PSN has been extensively studied.

### Keyword analysis

**Figure 7:**
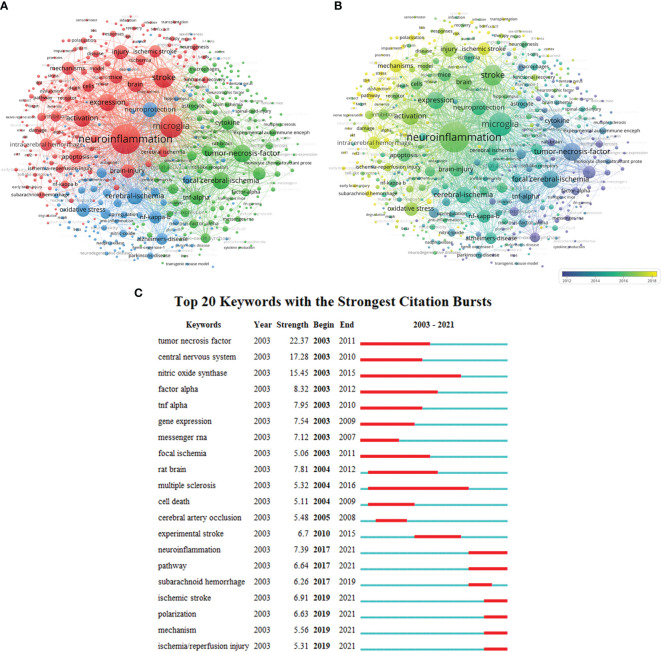
Co-occurrence network analysis of keywords regarding TNF in PSN study. **(A)** The keywords were divided into 3 categories according to different colors: category 1: red; category 2: green; and category 3: blue, where the size of the nodes indicates the frequency of occurrence; **(B)** Visualization of the keyword co-occurrence network according to the average years of publication. Keywords in yellow appear later than those in blue; **(C)** Top 20 keywords for burst detection.

The keywords of 1391 publications (including Author Keywords and Keywords plus) were analyzed in VOSviewer for keyword co-occurrence (**Figure 7**). The results indicated that Cluster 1 mainly concentrated on the study of PSN and microglia mechanisms while Cluster 2 focused on the mechanisms of cytokines such as TNF and PSN (**Figure 7A**). The Cluster 3 focused on the mechanisms of PSN and oxidative stress pathways, with the prevalent keyword being “stroke,” “expression,” “focal cerebral-ischemia,” “activation,” “oxidative stress,” “tumor-necrosis-factor,” and “mechanisms.” The keywords were color-coded into different types per the average year of publication (APY) utilizing the VOSviewer (**Figure 7B**). The areas such as neuroinflammation, mechanisms, rat model, and oxidative stress have become the main research areas in this field compared to older research keywords such as tumor-necrosis-factor and cytokines. The comparison of the two aforementioned figures (**Figures 7A, B**) shows that pathway and mechanism are the hot spots of research in neuroinflammation after stroke. The twenty keywords that aptly represent this field of research in terms of burst intensity, burst duration, and burst time were also examined (**Figure 7C**). In the early stages of PSN research areas such as tumor-necrosis-factor, central nervous system, and messenger RNA were the focus of research.

### Global citation score analysis

**Figure 8:**
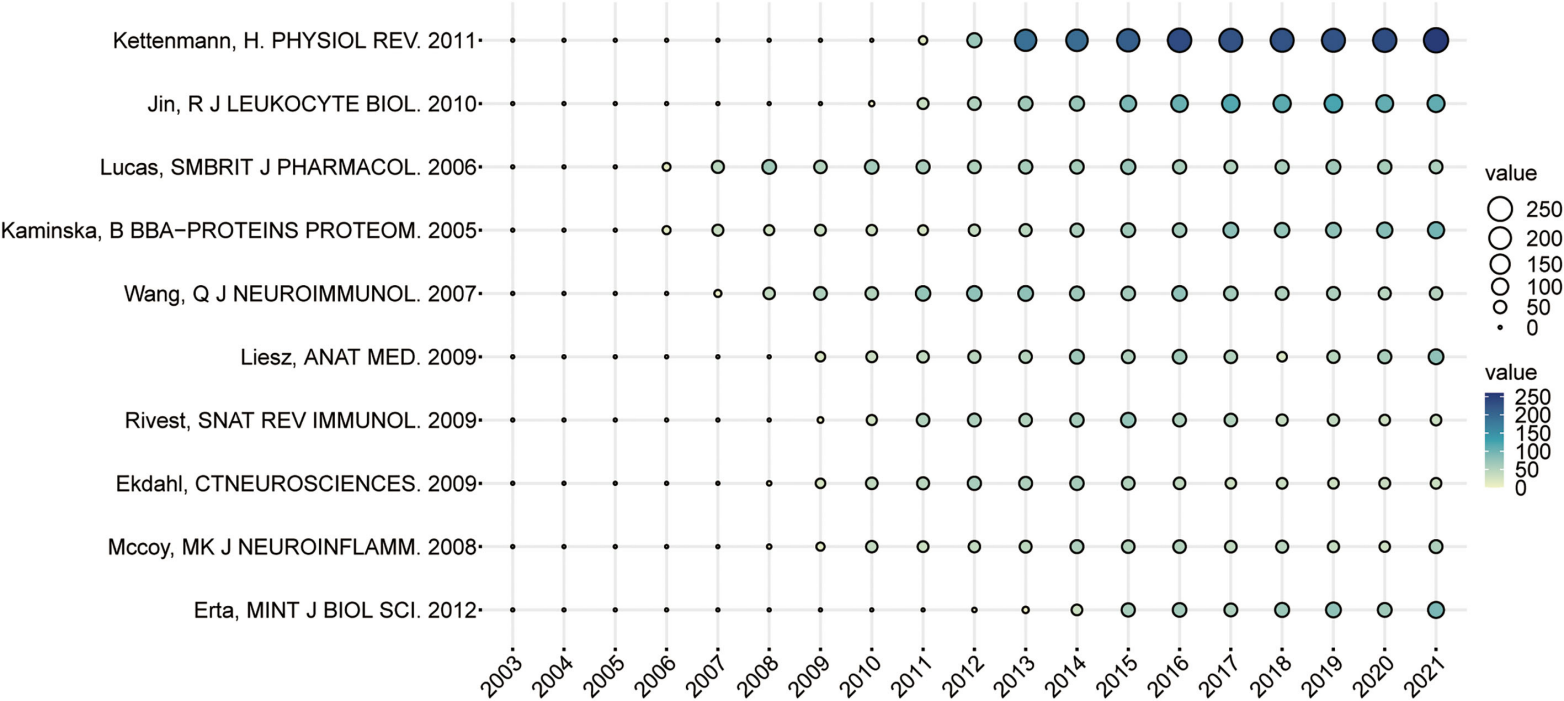
Annual global citations for papers with high GCS, where the size and color of the circles represent the GCS of the literature.

The top ten publications were analyzed regarding their global citation scores per year. And nine of the top ten publications were review, which is related to the relatively high impact and citation count of the high-level reviews. However, the important role of TNF in neuroinflammation due to stroke or other brain injury was highlighted in all nine reviews, and the TNF secreted by activated microglia plays an important role as an important inflammatory cytokine in various brain injuries, such as mediating the development of PSN and as a relevant target for effective treatment of PSN. Especially, the paper by Kettenmann (29), H published in 2011, ranked first with 2106 points (**Figure 8**) revealed the main mechanisms of PSN, that is once brain injury or neurological dysfunction occurs, microglia transforms into “activated microglia” and releases large numbers of substances such as TNF-α that are harmful or beneficial to surrounding cells, while migrating to the site of injury, proliferating and phagocytosing cells and cellular compartments. Lucas, S.M., et al. (30), highlighted the dual role of inflammatory mediators, including TNF-α, such as benefitting the long-term repair and recovery as well as being essential for the development of effective treatments for CNS disorders. TNF is reported to have both deleterious and protective actions in neurones, and these opposing effects may be explained by the existence of two distinct TNF-signaling pathways mediated by two receptors, p55 and p75. The author, Kaminska, B. et al. (31) systematically summarized current developments in the deleterious effects of inflammation, the regulation of TNF signaling pathways in cerebral ischemia, and potential molecular targets for anti-inflammatory therapy. Liesz, A et al. (32) proposed that Treg cell deficiency affected TNF-α (TNF-α) and interferon-γ (IFN-γ) expression in the brain by enhancing post-ischemic activation of inflammatory cells, including microglia and T cells, which increased delayed brain injury and worsened functional outcomes. Ekdahl, CT et al. (33) concluded that microglia activation as an indicator of inflammation is not per se pro- or anti-neurogenic and that its outcome depends on the balance between secreted molecules with pro- and anti-inflammatory effects, such as solTNF and tmTNF. The new and old observations from animal models and clinical trials reviewed by Mccoy, MK et al. (34) suggest that solTNF and tmTNF perform different functions in the CNS under normal and pathological conditions. SolTNF elevation is associated with acute and chronic neuroinflammation, as well as neurodegenerative conditions such as ischemic stroke. TmTNF is able to maintain immune functions such as self-tolerance and resistance to infection while limiting other functions of TNF and may selectively inhibit solTNF/TNFR1 signaling.

## Discussion

The current status and trends in the development of TNF-related research in PSN were analyzed for the first time ever by employing bibliometric analysis. According to the WoSCC database search, 1391 papers and reviews published from 2003 to 2021 were retrieved as of May 1, 2022. The spatial and temporal distribution, author contributions, and journal quality of these 1391 articles were assessed by employing CiteSpace and VOSviewer. In addition, burst hotspot analysis, cluster analysis, and keyword analysis were used to identify the currently more studied areas of research and frontiers of TNF-related research in PSN.

The change in annual trends based on published publications indicates a dynamic change from 12 annual publications in 2003 to 170 publications in 2021. Although the annual number of publications decreased in 2015 and 2017, studies on TNF in PSN have increased year by year. In this study, we also identified changes in TNF in PSN research over time: initially focusing on the key pro-inflammatory role of TNF in PSN, then focusing more on the specific signaling pathways of TNF-mediated inflammation in PSN, and more recently shifting to the therapeutic targets associated with the tmTNF-TNFR2 axis in PSN. We also predict that future research will be directed at how to better treat stroke in PSN by effectively balancing the biphasic effects of TNF. Therefore, the development of research on TNF in PSN is steadily progressing and improving.

An analysis of the top ten countries in terms of publications (**Figure 3B**) showed that the number of publications in China and the United States had increased each year since 2003, indicating the rapid development of TNF-related research in PSN in these two countries. China had the highest number of publications between 2003 and 2021, with 114 articles published in 2021. as shown in **Figure 4B**. Thus, China has a significant influence and is entering a phase of rapid development in the study of TNF in PSN. Papers published in the United States have the highest number of citations, indicating the high quality of publications published in the United States in this research area and a good reference value. The visual analysis of the cooperation between countries shows that the cooperation between China and the USA are closer, while the previous cooperation between other countries is less. In order to better carry out research and attack problems in this research area, it is strongly recommended that scholars from all countries break the academic boundaries and actively communicate and cooperate.

The analysis of the top ten institutions and authors of publications in this research area given in **Table 2** found that the European Research University Consortium has the highest number of publications, citations, and H-index, indicating its important role in this research area. Among the top ten institutions in terms of publications, 50% are Chinese universities or institutes and 40% are from the United States, indicating that China and the United States have a greater influence in this research area. The volume of publications and collaborations among institutions has also been illustrated (**Figure 4A**). Loma Linda University has the highest outbreak intensity, indicating that this institution may have had a larger output and contribution in this research area in the last five years (**Figure 4B**). To identify the most prolific authors, the top ten authors with the most publications were analyzed. Ranked first in this topic research area, Zhang, John H. from Loma Linda University ranked first in the field of TNF-related research on PSN, followed by Tang, Jiping from Loma Linda University and Finsen, Bente from University of Southern Denmark. The visual network of authors (**Figure 4C**) suggests less collaboration between authors and no network among principal investigators. It is suggested that more academic exchanges between scholars effectively increase collaboration to accelerate the progress of research in this field.

The publications published in this research area were counted using VOSviewer and 1391 articles published in 367 journals were assessed with publications in Journal of Neuroinflammation (IF: 9.587, JCR:Q1) ranking first in this research area. Among the top ten journals, only two journals (Journal of Neuroinflammation and Journal of Cerebral Blood Flow and Metabolism) have an impact factor of more than 6 points, indicating that no deeper or groundbreaking studies can be published in this research area. In addition, two journals were cited more than 3000 times, with Stroke (IF: 10.17, JCR:Q1) having the most citations, indicating the higher quality and better reference value of this journal in this field of study.

The two-plot overlay of journals shows the distribution of TNF in PSN studies across journal fields and the citation relationship between different journal fields. There are more studies related to PSN published in the Molecular/Biology/Genetics field. The study published in Journal of Neuroinflammation by Chen, S., et al., in 2017 (35) focused on ischemic stroke caused by elevated Hcy levels, which can effectively inhibit STAT3 phosphorylation by blocking the expression of JAK2/STAT3 signaling pathway as well as reducing the secretion of IL-6 and TNF-α, thereby reducing the occurrence of PSN. In addition, articles related to this study have been published in other fields, such as Physics/Materials/Chemistry, Veterinary/Animal/Science, Medicine/Medical/Clinical, and Neurology/Sports/. In the field of Veterinary/Animal/Science, articles related to this study focus on the specific mechanisms of neuroinflammation regulation after stroke through animal experiments, and in Psychology/Education/Health, research has focused on a range of psychological problems that result from post-stroke. It is worth noting that, in the field of Medicine/Medical/Clinical, studies related to this topic are mainly focused on clinical trials of drugs. For example, an article published by Brain Behavior and Immunity focuses on a natural product (36), pinocembrin, which has been shown to improve neuroinflammation and effectively reduce the expression of inflammatory factors such as IL-6,TNF-α in the brain tissue of patients with ischemic stroke. The pinocembrin may be a promising new drug candidate for the treatment of cerebral hemorrhage and other acute brain injuries. And in Neurology/Sports/Ophthalmology the specific pathogenesis and key regulatory pathways of PSN were mainly explored. The article published in Stroke analyze the biphasic role of microglia in stroke (37): microglia can play different roles in neuroinflammation by secreting different types of TNF-α through binding to TNFR1 or TNFR2, while microRNAs were found to play a biphasic role in post-stroke inflammation by regulating glial cell expression.

The reference co-citations facilitate the discovery of the knowledge base of the relevant research areas. The co-citations of references, with the top ten references cited, were also examined (**Figure 6A**). Among them, the most cited article is the original article “The immunology of stroke: from mechanisms to translation” (24) published by Iadecola C. et al. in the journal Nature Medicine in 2011. The aforementioned article focused on the relevant role between the immune system with post-ischemic brain tissue and the reduction of PSN by modulating adaptive immunity to reduce the levels of inflammatory factors such as TNF-α, offering the prospect of new therapeutic options for stroke. This article is highly cited literature and it’s probably because of the larger relevance of the main background knowledge of TNF in PSN in this research field to the issues studied in this paper.

Burst detection analysis can explore the development and evolutionary dynamics of a discipline’s research base and reveal sudden increases in popular citations in this research area over time. The 2010 article in Journal of Leukocyte Biology (38), which explores the mechanisms of inflammatory cell (inflammation) formation after ischemic stroke. The article, as a breaking hotspot, has a crucial role in addressing related issues and suggesting directions for research of PSN in the next step. A study on microglia/macrophage polarization kinetics published in Stroke in 2012 (39) found that under ischemic brain injury, the microglia/macrophages can change from an M2 phenotype, which favors brain tissue, to an M1 phenotype, which exacerbates brain tissue damage. This finding, as a burst hotspot, led to a research direction focused on the phenotypic transformation of microglia/macrophages. Since then, numerous studies have emerged to modulate microglia/macrophage polarization through therapeutic approaches such as drugs. The 2015 article published in the Journal of Neuroinflammation (40) found that Malibatol A. (MA), mice treated with a novel natural antioxidant extracted from the Chinese plant Hopea hainanensis showed decreased infarct size and brain damage after the middle cerebral artery occlusion (MCAO), effectively reducing ischemic brain injury as well as neuroinflammation, making it a potential therapeutic agent for stroke treatment. This finding has evolved in the last five years. Discoveries related to the extraction of natural antioxidant substances from plants to exert their anti-inflammatory effects and as novel drugs for the treatment of PSN have started to become a hot research topic.

Cluster analysis can be used to identify the main research topics, frontier directions, and progress in this research area by analyzing different clusters. A typical cluster analysis of the valid references of 1391 cited articles was performed to identify 19 homogeneous clusters of highly cited literature related to the study of TNF in PSN.

Cluster #16 (Neuropathology), which explores the pathophysiological changes in brain tissue after stroke, shows that inflammation is considered to be an important factor in the pathophysiology of ischemic stroke (41). According to the article related to cluster #5 (hypoxia), ischemic stroke leads to varying degrees of ischemia in brain tissue further leading to neuronal hypoxia in brain tissue, allowing microglia to receive chemoattractive and activating signals from injured neurons. The subsequent inflammatory transcriptional machinery begins to synthesize and release a large number of effector molecules such as pro-inflammatory cytokines. The production of pro-inflammatory cytokines, a common phenomenon in post-ischemic microglia, has received much attention in recent years. Many studies have suggested that pro-inflammatory cytokines are the main cause of secondary neuronal cell death. TNF-α is one of the most prominent pro-inflammatory cytokines. According to the analysis of cluster #0 (cytokines) and cluster #12 (inflammatory cytokines), both cytokines, TNF-α and IL-1β, exacerbate ischemic brain injury, while their inhibition reduces infarct volume (7, 42, 43). In most ischemic models, TNF-α was observed to be upregulated in post-ischemic microglia (44–48). Among the receptors of TNF-α produced by activated microglia, TNF-R1 (45, 49) and TNF-R2 (45, 48) were also reported to be upregulated after ischemia. The literature in cluster #1 (activated astrocytes) is dominated by studies on the effects and specific mechanisms of various types of glial cells on brain tissue during cerebral hemorrhage or other types of brain injury. It has been traditionally believed that reactive astrocyte proliferation and glial scar formation during ischemia are detrimental to recovery through physical and chemical inhibitory properties (50–52), however further studies have found that astrocyte proliferation is associated with neuroprotection (53, 54). Thus, astrocytes may play a dual role in the evolution of ischemic brain injury, with their ultimate impact strongly dependent on their interactions with microglia and neurons (55, 56) and polarization toward specific phenotypes (57). In the analysis of specific mechanisms, TNF also plays a different role as an indispensable cytokine. The TNF-α is mainly produced by activated microglia and enhances the tolerance of cultured neurons and astrocytes to oxidative stress and ischemic injury. Thus, microglia may promote neuroprotective functions against cerebral ischemia *via* TNF-p55R. This task will pave the way for the development of intervention strategies targeting microglia-produced mediators for the treatment of ischemic stroke.

One of the specific mechanisms *via* which TNF-α plays a role in post-ischemic brain injury was identified in cluster #17 (COX-1). The upregulation of cyclooxygenase (COX)-2 exacerbates neuronal injury after cerebral ischemia and leads to neuronal cell death (58). The peroxisome proliferator-activated receptor (PPARγ), which is primarily associated with anti-inflammatory processes, effectively inhibits (COX)-2 production and plays an important anti-inflammatory role in the post-ischemic inflammatory response. TNF-α is a potent suppressor of PPARγ expression in adipocytes, and this antagonism also appears to occur in inflammatory cells (59). This study found that TNF-induced microglial cell activation leads to a dramatic decrease in the number of PPARγ-positive cells (60).

The continuous development and refinement of research on TNF in PSN have led to further evolution of clusters, from the study of the specific roles and mechanistic pathways played by TNF in PSN to the investigation of the regulation of TNF to treat PSN and ensure a good prognosis. In terms of cluster #9 (TNF) and cluster #15 (microRNA), TNF can be expressed in brain tissue in two forms: a 26 kDa membrane-anchored form called transmembrane TNF (mTNF) and soluble TNF (solTNF), and a 17 kDa soluble form, which is mTNF after cleavage by the metalloprotease TNFα-converting enzyme TACE (61). Both TNFs further mediate different cellular functions through two TNF receptors (TNFR1 and TNFR2) (62–64), respectively. A study was conducted to investigate the role of TNF in PSN by using transgenic mice to alter the expression levels of both TNFs in mice. The results showed that elimination of solTNF while TNF maintained mTNF had a neuroprotective effect on focal cerebral ischemia (65). This finding may have implications for stroke treatment, as recently developed TNF inhibitors, such as XPro1595, can selectively target solTNF. Similar to the successful use of XPro1595 in the treatment of experimental spinal cord injury, local injection of XPro1595 after stroke may help to reduce lesion volume and inflammation and improve functional outcomes. Current research on TNF in PSN has focused on drug development, focusing on effectively improving the development of neuroinflammation by inhibiting or promoting the expression of a certain TNF, which is related to the articles covered in cluster #8 (Stroke Recovery). Per the findings of cluster #3 (TPA) (66), a better understanding of the spatiotemporal evolution of the inflammatory response associated with ischemic events and its potential role in later repair and regeneration processes is crucial for the development of novel and effective anti-inflammatory agents.

In addition to playing an important role in PSN, TNF plays other functions in the central nervous system. Related articles covered in Cluster #7 (CNS) and Cluster #18 (Memory) found that TNF is constitutively expressed in the brain and exerts important physiological functions as a regulator of neuronal activity. Neurotransmitter modulation produces glial-derived TNF that plays a necessary role in synaptic scaling and synaptic strength preservation (67, 68). In addition, a normal cognitive function under initial conditions relies on TNF and its receptors deficiency of TNF improves spatial learning and memory and reduces anxiety levels in mice (69), whereas knockdown of TNF receptor-increase exploration and anxiety-like behavior (70, 71).

The keyword co-occurrence analysis and keyword burst intensity analysis can be used to explore the research direction and hot frontier of this field. In the analysis of keyword burst intensity, the keyword “TNF” appeared most frequently in this field, followed by the “central nervous system.” This is related to the fact that the research on TNF was a hot spot in this field during 2003-2011. Analysis of relevant articles appearing from 2003 to 2011 revealed that most of them mainly explored the mechanisms and specific roles of TNF-α as an inflammatory cytokine in the central nervous system in acute brain injury. However, new research directions have emerged in the past five years, and studies on TNF-related issues have focused on the analysis of specific signaling pathways of TNF in neuroinflammation, and more conclusions and findings have been obtained from the research on the transcriptional translation of TNF in various glial cells and secretion through vesicles.

Meanwhile, TNF-R1 and TNF-R2 receptor regulation, which are involved in the activation of TNF in neuroinflammation, have also become a recent hot topic of research. The prevailing therapeutic approach is to selectively inhibit solTNF/TNFR1 signaling to improve the prognosis of brain injury by reducing the production of pro-inflammatory signals while preserving the function of the tmTNF/TNFR2 axis. Thus, selective blockade of solTNF by genetic ablation or the soluble inhibitor XPro1595 reduces neuroinflammation in experimental models of MS (72) and ischemic stroke (65, 73). Fewer treatment options target the tmTNF/TNFR2 axis. One study used a selective TNFR2 agonist, which showed protective effects in a model of NMDA-induced acute neurodegeneration (74). Meanwhile, another study found that TNF promotes re-sheathing in oligodendrocyte growth and suppresses autoimmunity simultaneously, by promoting tmTNF/TNFR2 simultaneously, and has a protective effect in demyelinating disease, and improve motor and cognitive symptoms in EAE mice effectively (75). The analysis revealed that the future research prospect may be mainly reflected in the regulation of solTNF-TNFR1 axis/tmTNF-TNFR2 axis to treat PSN and improve its prognosis more precisely and effectively.

## Conclusion

With the help of CiteSpace and VOSviewer, a deeper understanding of the research development, hotspots and future trends of TNF in PSN over 19 years has been gained. Leading countries are China and the USA, but countries, institutions, and authors need to collaborate and communicate better. The bibliometric analysis provides an objective and quantitative method to assess the trends and frontiers of TNF in PSN, and also provides important clues for researchers to understand the structural and temporal dynamics of the field. The current study also summarizes the bidirectional roles of TNF in PSN and reveals that the future research prospect may be mainly reflected in the regulation of bidirectional pathway of TNF to treat PSN.

## Data availability statement

The raw data supporting the conclusions of this article will be made available by the authors, without undue reservation.

## Author contributions

YZ and QZ collected the data, wrote the paper. YZ and CB analyzed the data, performed the data curation. XH, YC and JY revised the paper. YZ and QZ contributed equally to this work. All authors contributed to the article and approved the submitted version.

## Funding

This work was supported by grants from Joint Medical Research Project of Chongqing Science and Technology Commission and Health Commission (2021MSXM262) and Chongqing Natural Science Foundation (2022NSCQ-MSX4507).

## Acknowledgments

The authors acknowledge gratitude to all the staff who participated in this study.

## Conflict of interest

The authors declare that the research was conducted in the absence of any commercial or financial relationships that could be construed as a potential conflict of interest.

## Publisher’s note

All claims expressed in this article are solely those of the authors and do not necessarily represent those of their affiliated organizations, or those of the publisher, the editors and the reviewers. Any product that may be evaluated in this article, or claim that may be made by its manufacturer, is not guaranteed or endorsed by the publisher.
